# PLoS Currents: Evidence on Genomic Tests – At the Crossroads of Translation

**DOI:** 10.1371/currents.RRN1179

**Published:** 2010-09-02

**Authors:** Marta Gwinn, W. David Dotson, Muin J. Khoury

**Affiliations:** ^*^McKing Consulting Corp and ^†^Office of Public Health Genomics, CDC

## Abstract

Evidence on Genomic Tests is an open access publication option for communicating high-quality, scientific information that is needed to evaluate health applications of genomic research. By using Google’s knol platform, we aim to reduce conventional barriers to sharing, updating, and accessing the results of knowledge synthesis and to increase the benefits to authors and users alike.

      Good, fast, and affordable technologies are driving the “genomics revolution” in biomedical research. The Internet serves as an amplifier, rapidly publishing research findings and publicizing them to a wide audience. Public interest in the field has focused on potential health applications, especially genomic tests for predicting, diagnosing, and managing common diseases.  Although many such tests are emerging, information on their clinical validity and utility tends to be scattered and incomplete.

      Today we are taking advantage of an innovative, online publication format to launch ***PLoS Currents: Evidence on Genomic Tests ***as an open access publication option for communicating high-quality, scientific information that is needed to evaluate health applications of genomic research. By using Google’s *knol* platform, we aim to reduce conventional barriers to sharing, updating, and accessing the results of knowledge synthesis and to increase the benefits to authors and users alike. A few months ago, we ‘soft-launched’ a version of this publication called Evidence for Genomic Applications, but now we are joining forces with the Public Library of Science (PLoS), which has pioneered this approach to publish *PLoS Currents: Influenza*. In this model, new submissions are assessed by a board of expert reviewers; articles are published immediately on approval; and each publication is given a unique identifier and archived in PubMed Central. The new section of PLoS Currents that we are launching is called *PLoS Currents: Evidence on Genomic Tests*.

      ***PLoS Currents: Evidence on Genomic Tests ***is intended to complement other efforts to evaluate genomic applications, both ongoing and planned, by using carefully targeted approaches. Wewill publish brief, structured summaries of essential evidence for the validity and utility of genomic tests. These summaries may be based on completed, in-depth, systematic reviews, such as those produced by the  Evaluation of Genomic Applications in Practice and Prevention (EGAPP) project[1] or the U.S. Preventive Services Task Force (USPSTF) that have been published elsewhere, supplemented with additional information that may have appeared since publication. Alternatively, summaries may focus on genomic tests for which no systematic review has been completed, often because the available evidence is considered too incomplete and fragmentary to warrant it. For such tests, ***PLoS Currents:*** ***Evidence on Genomic Tests ***will publish summaries of the most relevant available information, including key links, and will highlight critical knowledge gaps. 

      The term ‘Genomic Tests’ was selected for use in our title, rather than ‘Genetic Tests’, because we seek to include a broad array of genomic technologies, such as proteomics and epigenetics, that are applied not only in the diagnosis of heritable disorders, but in predicting predicting, diagnosing, and managing common diseases. Most authors of articles published in ***PLoS Currents:*** ***Evidence on Genomic Tests ***will be researchers in genetics, knowledge synthesis, or related fields. In addition to researchers, potential readers include public health and health care practitioners and decision-makers. The *knol* format includes several innovative capabilities, such as tagging, rating, and monitoring activity; it also allows readers to comment, which creates a new opportunity for feedback from knowledge users to knowledge providers. In contrast to the open wiki model, however, readers are not able to edit published articles, and all comments will be identified and reviewed by the moderators before posting. 

      The Human Genome Project raised expectations that a new generation of genetic tests would soon be available to predict and diagnose a wide array of health conditions.[2] Even before large-scale genome sequencing began, the National Institutes of Health (NIH)-Department of Energy (DOE) Working Group on Ethical, Legal, and Social Implications of Human Genome Research created a task force to make recommendations to ensure the development of safe and effective genetic tests in the United States. The Final Report of the Task Force on Genetic Testing, published in September 1997, recommended that before new genetic tests became available for non-investigational use, they should be reviewed at the national level “by professional societies, consensus panels, Federal agencies and other organizations.” The task force report defined the basis for evaluation in terms of analytic validity, clinical validity, and clinical utility. These concepts continue to provide the foundation for evaluating genomic tests.  

      In the absence of regulation requiring formal review and approval, several groups have worked to develop methods for systematically reviewing the validity and utility of genomic tests. For example, from 2000 to 2004, the U.S. Centers for Disease Control and Prevention (CDC) sponsored the ACCE project, which developed a model process for evaluating scientific data on emerging genomic tests.[3] The EGAPP initiative has further developed and applied this model by publishing recommendations based on systematic reviews of genomic tests in transition from research to clinical and public health practice. The USPSTF has also released recommendations on specific genomic tests used in selected clinical scenarios. Evidence reviews for recommendations such as these have been conducted by Evidence-based Practice Centers sponsored by the Agency for Healthcare Research and Quality, as well as by other groups.

      In 2008, a the Secretary’s Advisory Committee on Genetics, Health, and Society Task Force on Oversight of Genetic Testing recommended that “to enhance the transparency of genetic testing and assist efforts in reviewing the clinical validity of laboratory tests, the U.S. Department of Health and Human Services should appoint and fund a lead agency to develop and maintain a mandatory, publicly available, Web-based registry for laboratory tests.”[4] A subsequent article outlined a “blueprint” based on these recommendations, suggesting that registration should be mandatory for clinical laboratories and other businesses offering these tests to health care providers (or in some cases, directly to consumers).[5] NIH has since announced its intention to establish a voluntary Genetic Testing Registry by 2011.[6]

      Whether mandatory or voluntary, implementation of a genomic test registry faces major challenges in implementation, particularly regarding the sources and quality of submitted data and their systematic evaluation. Laboratories that perform genomic tests are likely to have only some of the data needed to evaluate clinical validity and utility. A comprehensive assessment will require bringing together data from multiple sources—including basic research, clinical trials, and epidemiological and clinical studies—for systematic review.[7]

      ***PLoS Currents: Evidence on Genomic Tests ***provides a forum for sharing credible data, with the goal of making it actionable. Of course, assessing scientific evidence is only one dimension of research translation, which requires interactions among many sectors of society. This new publicationwill make evidence summaries accessible to a broader array of stakeholders via links from other online resources, like the GAPPNet Knowledge Base, a component of the Genomic Applications in Practice and Prevention Network (GAPPNet) recently convened by CDC and the National Cancer Institute. Making use of the Internet’s capacity for connection will help ***PLoS Currents: Evidence on Genomic Tests ***monitor the busy crossroads of research translation. 
